# Angiotensin-converting enzyme inhibitor for post-transcatheter aortic valve implantation patients: study protocol for a multicenter randomized, open-label blinded endpoint control trial

**DOI:** 10.1186/s13063-021-05411-5

**Published:** 2021-07-18

**Authors:** Yan Biao Liao, Congying Xia, Yiheng Cheng, Qiao Li, Xin Wei, Yuanweixiang Ou, Fei Chen, Yijian Li, Qi Liu, Tianyuan Xiong, Zhengang Zhao, Yong Peng, Jiafu Wei, Yuan Feng, Mao Chen

**Affiliations:** Department of Cardiology, West China Hospital, Sichuan University, Chengdu, China

**Keywords:** Aortic stenosis, Fosinopril, Left ventricle mass, Renin-angiotensin system inhibitor, Prognosis

## Abstract

**Background:**

With the expanded utilization of transcatheter aortic valve implantation (TAVI) to younger and lower surgical risk patients with severe aortic stenosis (AS), optimal medical therapy after TAVI procedure has become the main concern. Renin-angiotensin system inhibitors (RASi) are widely utilized in the area of cardiovascular disease including heart failure and myocardial infarction and revealed the ability to reverse left ventricular (LV) remodeling. Interests have, thus, been drawn in investigating whether the prescription of RASi after the TAVI procedure can prevent or reverse cardiac remodeling and improve long-term clinical outcomes. No recommendation regarding the prescription of RASi after TAVI is proposed yet due to the lack of evidence from randomized controlled trials, especially in the Chinese population. We, therefore, designed this randomized controlled trial to explore the effect of adding fosinopril to standard care in patients who underwent a successful TAVI procedure on the LV remodeling.

**Methods:**

A total of 200 post-TAVI patients from seven academic hospitals across China will be recruited and randomized with a ratio of 1:1 to receive standard care or standard care plus fosinopril. Follow-up visits will take place at 30 days, 3 months, 6 months, 12 months, and 24 months from randomization to assess the clinical symptoms, any adverse events, cardiac function, and quality of life. Cardiac magnetic resonance will be performed at baseline and repeated at the 24-month follow-up visit to assess LV remodeling.

**Discussion:**

This study will provide evidence regarding medical therapy for AS patients who underwent TAVI and filling the gap in the Chinese population.

**Trial registration:**

Chinese Clinical Trial Registry ChiCTR2100042266. Registered on 17 January 2021

**Supplementary Information:**

The online version contains supplementary material available at 10.1186/s13063-021-05411-5.

## Administrative information

Note: the numbers in curly brackets in this protocol refer to SPIRIT checklist item numbers. The order of the items has been modified to group similar items (see http://www.equator-network.org/reporting-guidelines/spirit-2013-statement-defining-standard-protocol-items-for-clinical-trials/).

**Table:** 

Title {1}	Angiotensin-converting enzyme inhibitor for post transcatheter aortic valve implantation patients: study protocol for a multicenter randomized, open-label blinded endpoint control trial
Trial registration {2a and 2b}.	This study was registered at the Chinese Clinical Trial Registry (http://www.chictr.org.cn), 17 January 2021. The registration number is ChiCTR2100042266.
Protocol version {3}	Version 1.1
Funding {4}	This randomized trial is funded by a grant from the Chinese Association of Cardiology (CSCF2020B04).
Author details {5a}	YanBiao Liao, Congying Xia, Yiheng Cheng, Qiao Li, Xin Wei, Yuanweixiang Ou, Fei Chen, Yijian Li, Qi Liu, Tianyuan Xiong, Zhengang Zhao, Yong Peng, Jiafu Wei, Yuan Feng, Mao Chen Authors are all from Department of Cardiology, West China Hospital, Sichuan University, Chengdu, China
Name and contact information for the trial sponsor {5b}	Chinese Medical Association Cardiovascular Branch (CSC) Clinical Research Special Fund Project. Yinghong Ai, +8613940094018, xlzc@cardiounion.cn
Role of sponsor {5c}	This funding source had no role in the design of this study and will not have any role during its execution, analyses, interpretation of the data, or decision to submit results.

## Introduction

### Background and rationale {6a}

Aortic stenosis (AS) is the most common valvular heart disease in the elderly population [1]. With the acceleration of the Chinese population aging, the prevalence of aortic stenosis is thought to increase markedly. Surgical aortic valve replacement (SAVR) is regarded as the only effective method for patients with aortic stenosis in the past decades. However, numerous evidence have demonstrated transcatheter aortic valve implantation (TAVI) to be comparable and sometimes superior in treating patients with aortic stenosis regardless of risk stratification, in comparison with SAVR [2].

Patients with aortic stenosis typically accompany with left ventricular (LV) hypertrophy or fibrosis indicating a worse long-term clinical outcome [3]. A majority of patients continue to present left ventricular hypertrophy even after SAVR or TAVI, who had a high risk for mortality and heart failure re-admission [4]. A previous meta-analysis found that the 30-day and 1-year incidence of re-admission following TAVI was 15% and 31%, respectively, of which nearly half resulted from the cardiac cause [5]. Compared with that of SAVR, TAVI is associated with a high risk for paravalvular leakage, even in patients with a new-generation transcatheter heart valve. The persistent left ventricular hypertrophy and new-onset paravalvular leakage after TAVI will negatively affect the prognosis especially when TAVI moves toward to treating patients at a young age.

Renin-angiotensin system inhibitors (RASi) are widely utilized in the area of cardiovascular disease including heart failure, myocardial infarction, and so on due to the ability to reversing the left ventricular remodeling [6, 7]. Candesartan was documented to be associated with positive improvement in left ventricular remodeling in patients with preserved left ventricular ejection fraction (LVEF) after SAVR in a randomized controlled trial [8]. Similarly, several retrospective studies also demonstrated RASi could reduce the occurrence rate of heart failure re-admission and mortality in patients with aortic stenosis after SAVR and TAVI [9–11]. However, no recommendation regarding the prescription of RASi after TAVI was proposed yet due to lacking randomized controlled trials.

Therefore, we designed this randomized controlled trial (http://www.chictr.org.cn ChiCTR2100042266) to explore the effect of adding fosinopril to standard care in patients with preserved LVEF following successful TAVI procedure on the left ventricular remodeling and fibrosis as evaluated by cardiac magnetic resonance (CMR) and echocardiography.

### Objectives {7}

The primary objective of this trial is to investigate the efficacy of RASi in the improvement of left ventricular remodeling quantified by CMR in patients after TAVI. The secondary objective is to assess potential improvements in clinical outcomes including all-cause mortality, cardiac-cause mortality, heart failure status assessed by the New York Heart Association (NYHA) class, and 6-min walk test (6MWT), and quality of life as assessed by the Kansas City Cardiomyopathy Questionnaire (KCCQ) score.

### Trial design {8}

This study will be a prospective, randomized, open-label blinded endpoint control trial in symptomatic patients with severe AS. This study protocol was approved by the institutional ethics committee of West China Hospital of Sichuan University and the local medical ethics committee of each participating center.

## Methods: participants, interventions, and outcomes

### Study setting {9}

This trial will take place at seven academic medical centers across China. The list of the involved hospitals is presented in supplemental table 1.

### Eligibility criteria {10}

Symptomatic patients with severe AS after the TAVI procedure will be randomized to receive either conventional treatment or conventional treatment plus fosinopril. Severe AS is determined according to echocardiography measures: (1) aortic valve area ≤ 1.0 cm^2^ or aortic valvular area index ≤ 0.6 cm^2^/m^2^ and (2) peak aortic jet velocity ≥ 4 m/s or mean transaortic valvular pressure gradient ≥ 40 mmHg.

#### Inclusion criteria

Subjects need to meet all the following inclusion criteria to participate in the study: (1) men or women aged ≥ 60 years, (2) presenting with symptomatic severe AS, (3) have undergone TAVI successfully which have to be approved by the local heart team according to the Valve Academic Research Consortium-2 consensus document [12], (4) heart failure is classified as function capacity level I according to the NYHA, (5) LVEF ≥ 40% assessed by echocardiography, and (6) being able to understand and provide written informed consent.

#### Exclusion criteria

Subjects presenting any of the following exclusion criteria will be excluded from the study: (1) concomitant with other significant valvular heart disease needs intervention; (2) contraindication or intolerance for the use of fosinopril including a history of angioneurotic edema, hypotension (defined as systolic blood pressure < 100 mmHg or diastolic blood pressure < 60 mmHg), estimated glomerular filtration rate < 30 mL/min/1.73 m^2^, and random serum potassium > 5.4 mmol/L; (3) life expectancy < 1 year; (4) contraindication to CMR, f.e., implantation of cardiac resynchronization therapy devices that are non-magnetic resonance conditional; and (5) prior history of myocardial infarction or dilated cardiomyopathy.

### Who will take informed consent? {26a}

Written informed consent will be collected from all participants. A researcher from the local heart team, who is trained with the study protocol will confirm the patient’s willingness to participate and eligibility criteria and obtain an informed consent.

### Additional consent provisions for collection and use of participant data and biological specimens {26b}

Not applicable

### Interventions

#### Explanation for the choice of comparators {6b}

Patients allocated in the control group will receive standard care according to the current practice.

#### Intervention description {11a}

Patients allocated in the intervention group will receive an additional use of fosinopril with an initial dose of 10 mg once daily. If the patient is well-tolerated, they will be titrated to a maximum dose of 40 mg once daily. Titration of fosinopril will be performed at the 1-month follow-up visit and afterward.

#### Criteria for discontinuing or modifying allocated interventions {11b}

Participants are allowed to discontinue and end their participation in the trial at any time for any reason. Researchers can decide to withdraw a participant from the trial at any time when encountering emergency circumstances and/or for any urgent medical reasons.

#### Strategies to improve adherence to interventions {11c}

A standard operating procedure was created for the administration of treatment of intervention and control group. Research assistants will help educate the patients. Adherence to the use of fosinopril will be assessed during follow-up visits.

#### Relevant concomitant care permitted or prohibited during the trial {11d}

In the case of patients allocated in the control group and who need to be treated for hypertension, the local Heart Team will make the decision without prescribing any RASi.

#### Provisions for post-trial care {30}

We expect that intervention-related severe adverse events in this trial will be unlikely because the use of RASi in patients with heart failure is a routine regimen in current practice. Nevertheless, we plan to provide health care at each study site for participants who may encounter trial procedure-related medical situations that need ancillary care. Post-trial care will not be provided.

#### Outcomes {12}

The primary outcome of this trial is the changes in LV mass index. The secondary outcomes include the following: (1) changes in LVEF; (2) all-cause mortality; (3) cardiac death; (4) re-admission rate due to heart failure; (5) changes in serum N terminal pro B type natriuretic peptide (NT-ProBNP), a diagnostic biomarker for heart failure; (6) NYHA class, KCCQ scores, and results of 6MWT at 24 months follow-up; and (7) changes in NYHA class, KCCQ scores, and results of 6MWT.

#### Participant timeline {13}

**Fig. 1:**
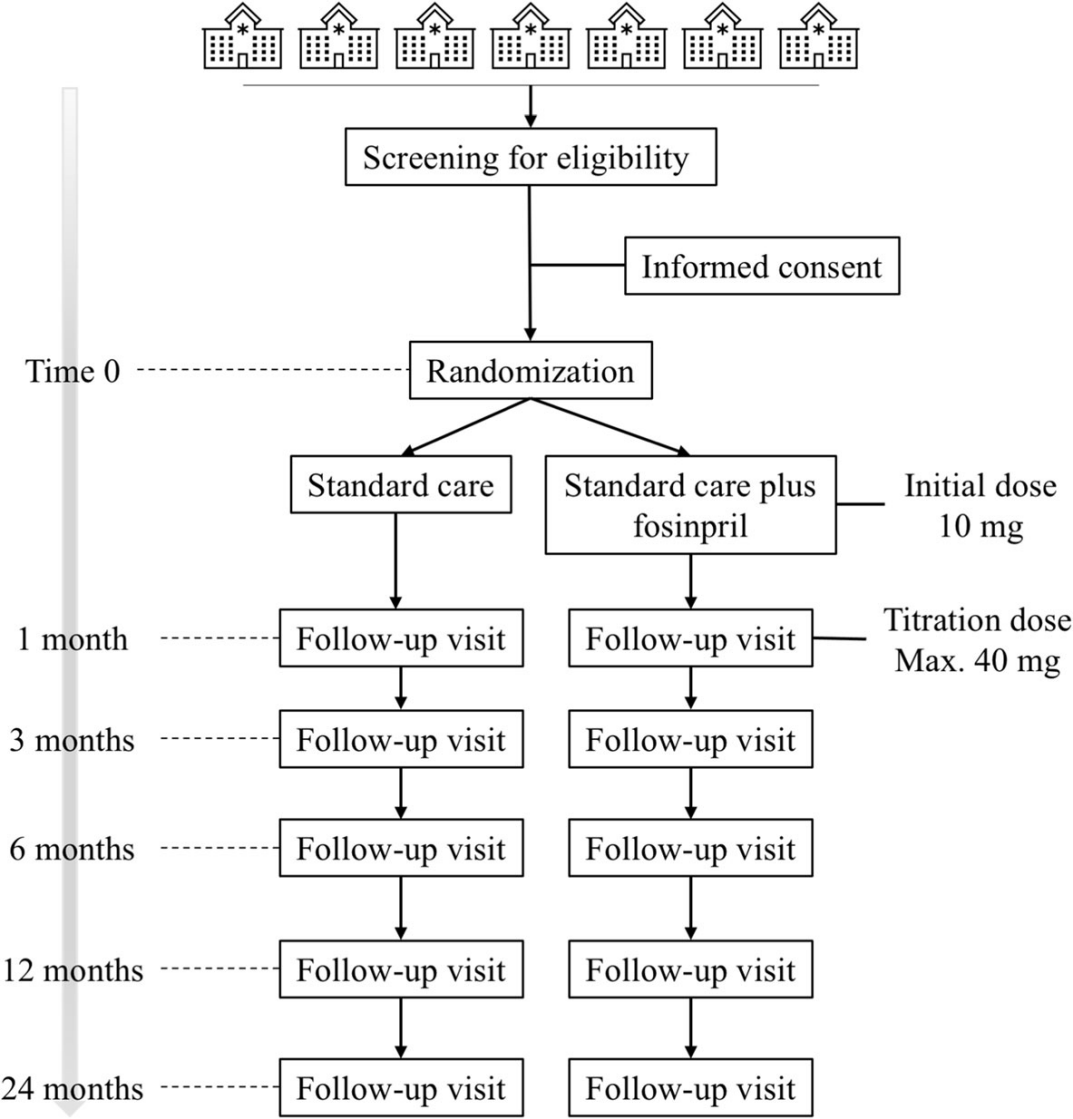
Flowchart of the study design

The workflow is depicted in the flowchart (Fig. 1); schedule of enrollment, intervention, and examinations are presented in Table 1.

**Table 1:** Schedule of enrollment, interventions, and examinations

Time point	Study period						
	Enrolment	Allocation	Post-allocation				
	1 week pre	0	1 month	3 months	6 months	12 months	24 months
Enrollment							
Eligibility screen	X						
Informed consent	X						
Allocation		X					
Interventions							
Standard care			X	X	X	X	X
Standard care plus fosinopril			X	X	X	X	X
Assessments							
Demographics	X						
Physical examinations	X		X	X	X	X	X
Medical history	X		X	X	X	X	X
Laboratory tests	X		X	X	X	X	X
NYHA class	X		X	X	X	X	X
Transthoracic echocardiography	X		X	X	X	X	X
CMR		X					X
KCCQ		X	X	X	X	X	X
6-min walk test		X	X	X	X	X	X
Adverse events			X	X	X	X	X

*NYHA* New York Heart Association, *CMR* cardiac magnetic resonance, *KCCQ* Kansas City Cardiomyopathy Questionnaire

#### Sample size {14}

The sample size is calculated based on the primary endpoint, namely the difference in changes of LV mass index at 24 months between the intervention group and the control group. Prior prospective studies reported a difference of 10 g/m^2^ and 18 g/m^2^ in absolute changes in LV mass index at 6 months and at 12 months, respectively, comparing with and without RASi treatment [8, 13]. A sample size of 100 per group will have more than 90% power to detect an expected difference of 20 g/m^2^ at an alpha level of 0.05, given a standard deviation of 35 g/m^2^ and a dropout rate of 10%.

#### Recruitment {15}

Patients undergoing TAVI from seven academic medical centers across China will be screened for eligibility and recruited about 1 week after the TAVI procedure.

### Assignment of interventions: allocation

#### Sequence generation {16a}

Eligible patients will be randomized in a 1:1 ratio to receive standard care or standard care plus fosinopril. Centralized random allocation will be performed after receipt of informed consent using a block size of 4 without stratification. Allocation sequence was generated using R with the package randomizeR prior to study initiation.

#### Concealment mechanism {16b}

Generated allocation sequence is to be concealed in opaque, sealed envelopes that are consecutively numbered. An independent research assistant is responsible for keeping these envelopes unopened and allocated.

#### Implementation {16c}

The allocation sequence will be generated by a statistician. Participants who are eligible for this trial and willing to give written consent will be enrolled by a trial researcher. The research assistant will directly inform the clinician about the group allocation.

### Assignment of interventions: blinding

#### Who will be blinded {17a}

Due to logistic reasons, it will not be feasible to blind the natural course of this study to patients nor clinicians. However, all endpoints will be adjudicated by an independent imaging core lab or the clinical event committee, who are blinded to the group allocation.

#### Procedure for unblinding if needed {17b}

Not applicable, the trial is an open-label design.

### Data collection and management

#### Plans for assessment and collection of outcomes {18a}

Severe AS patients who underwent TAVI will be approached and consented for screening for eligibility. Screening evaluations include brief physical examinations, blood laboratory tests, and reviewing of medical history. Heart failure-related symptoms will be assessed for an NYHA functional classification. Transthoracic echocardiography will be conducted to assess cardiac function.

The following information will be collected during baseline evaluations: (1) demographics including date of birth, gender; (2) physical examinations including body weight, body height, resting pulse, and blood pressure; (3) any major previous health problems and use of medication by reviewing health records; (4) NYHA function capacity class by examining related symptoms; (5) results of laboratory tests including full blood count, blood lipids, glucose, liver function, renal function, and NT-ProBNP; (6) KCCQ score and results of 6MWT. Follow-up visits will take place 30 days, 3 months, 6 months, 12 months, and 24 months from randomization. At each follow-up visit, patients will be asked for any specific symptoms, any changes in the use of medication, and any adverse events. A brief physical examination will be performed to measure the resting pulse and blood pressure. Blood samples will be taken for the laboratory tests including NT-ProBNP. Transthoracic echocardiography will be performed to evaluate LV function. Moreover, NYHA function capacity class, KCCQ, and 6MWT will be evaluated. CMR scanning will be performed at baseline and be repeated at the 24-month follow-up visit to assess LV volume, mass, and function. CMR images will be analyzed independently by a central imaging core lab (West China Hospital Radiology Medicine lab). All endpoints will be adjudicated by an independent clinical events committee blinded for group allocation.

#### Kansas City cardiomyopathy questionnaire

KCCQ is used to assess patients’ quality of life [14]. The KCCQ is self-administered, but patients can also complete the questionnaire by interview through a clinician. On average, it will take 4 to 6 min to complete the KCCQ.

#### Six-minute walk test

The 6MWT will be performed in an indoor hospital corridor at each participating site. The walking course is straight, flat, and at least 30 m in length. The starting line and every 3 m of the course are marked. Patients will be asked to walk along the corridor back and forth. Patients will be allowed to stop and rest or discontinue the test. The longest distance in 6 min will be measured to the nearest whole meter. In order to maintain consistency, all participating sites will use a standardized manual to guide operations, including standardized phrasing to give patients instructions and encouragement.

#### Transthoracic echocardiography protocol

Transthoracic echocardiography will be performed using commercially available ultrasound systems at each participating sites. In order to maintain consistency, all measurements will be performed according to the expert consensus on adult transthoracic echocardiography reporting [15].

#### Cardiovascular magnetic resonance protocol

CMR scanning will be performed using a 1.5-T scanner which is commercially available at local participating sites. An electrocardiography-triggering acquisition will be conducted for patients in supine position and breath-hold at the end of expiration. Late gadolinium enhancement CMR was also conducted by the administration of 0.2 mmol/kg of gadolinium. After scout imaging, the long axis of the LV and four-chamber cine images will be acquainted using the balanced steady-state free precession (bSSFP) sequence with a slice thickness of 8 mm and interslice gap of 2 mm. Imaging acquisition parameters are as follows: repetition time 34 ms, echo time 1.3 ms, flip angle 50°, average temporal resolution 35–45 ms, and field of view 280 × 340 mm^2^.

Images will be evaluated using the Cvi42 version 5.12 software (Circle Cardiovascular Imaging Inc.) for LV mass, volumes, and function. LV mass is calculated by multiplying the difference between total epicardial volume and total endocardial volume with the density of myocardium (1.05 g/mL). LV mass is then indexed to the surface area to achieve LV mass index [16].

#### Plans to promote participant retention and complete follow-up {18b}

All reasonable efforts (such as by email and telephone call) will be made to complete the data collection including follow-up information for endpoints (if applicable) and the reason why participants want to quit the trial.

### Data management {19}

A digital case reported form will be designed and used for data collection. All collected data will be stored and managed using a web-based data manage system. Researchers are responsible for the data collection, especially for checking that all data related to this trial are filled out in the data manage system correctly and completely. A data management plan will be created beforehand for data validation.

#### Confidentiality {27}

Personal data of the participants will be treated as confidential and stored securely during the whole course of this trial.

#### Plans for collection, laboratory evaluation, and storage of biological specimens for genetic or molecular analysis in this trial/future use {33}

Not applicable

## Statistical methods

### Statistical methods for primary and secondary outcomes {20a}

The primary and secondary endpoints will be analyzed according to the intention-to-treat principle. To compare the primary endpoint, adjusted mean difference in changes in LV mass index at 24-month follow-up visit between the two groups will be tested using linear regression with adjustment for baseline values. Time-to-event comparison (f.e., secondary endpoints including all-cause mortality and cardiac death) between the groups will be conducted using log-rank test and proportional hazards regression. A two-sided p value of 0.05 will be considered statistically significant.

### Interim analyses {21b}

Not applicable, no interim analysis will be performed.

### Methods for additional analyses (e.g., subgroup analyses) {20b}

For descriptive analyses of baseline characteristics, continuous variables will be presented as mean and standard deviation or median and interquartile range depends on the distribution. Categorical variables will be presented as frequencies and percentages. Differences between the groups will be tested using the independent t-test or Mann-Whitney U test when appropriate for continuous variables and using the chi-square test or Fisher’s exact test when appropriate for categorical variables.

### Methods in analysis to handle protocol non-adherence and any statistical methods to handle missing data {20c}

All efforts will be made to complete the follow-up and data collection and avoid missing data. Analyses will be conducted in complete cases. We will also perform sensitivity analyses by imputing the missing data to worst cases.

### Plans to give access to the full protocol, participant level-data, and statistical code {31c}

Statistic plan and statistical code will be published together with the final reports after the whole trial is closed. There is no plan to release the participant-level dataset; however, access to the dataset will be possible upon reasonable requisition.

## Oversight and monitoring

### Composition of the coordinating center and trial steering committee {5d}

The coordinating center is the West China Hospital. The trial steering committee consists of principal investigators at each study site responsible for interventions (standard care for the control group, standard care plus fosinopril for the intervention group). Other clinicians of the local Heart Team will be responsible for follow-up, data collection, and logistic support for the trial. The endpoint adjudication committee consists of an imaging core lab and two cardiologists.

### Composition of the data monitoring committee, its role, and reporting structure {21a}

The data and safety monitoring committee (DSMC) will consist of four qualified members from the West China Hospital, including at least one statistician and cardiology expert independent from the site trial staff. The DSMC is independent from the trial researchers, blinded to the assignment, and will monitor the progress of the trial and safety issues and require data for analyses.

### Adverse event reporting and harms {22}

Adverse events reported by the participant or noticed by the clinician will be reported directly (maximum within a week) to the DSMC and recorded in the digital case reported form and data manage system.

### Frequency and plans for auditing trial conduct {23}

Audit reports will be given by the trained auditors at three different time points, namely after the first participant is enrolled, at 12 months after launching the trial, and before the trial is closed out. Eligibility of recruited participants, collection of consent, and data integrity will be checked at each study site.

### Plans for communicating important protocol amendments to relevant parties (e.g., trial participants, ethical committees) {25}

A yearly update will be given to the ethics committee if any protocol amendment is supposed to be made in line with the updates for clinical practice. Amendments will be made only after a favorable opinion is given by the ethical committees. All site trial staff will be informed correspondently.

### Dissemination plans {31a}

The trial results will be presented at international meetings and published in a peer-reviewed journal.

## Discussion

With the expanded utilization of TAVI to a younger and lower surgical risk patient with severe AS, optimal medical therapy after TAVI procedure has become the main concern. Interests have been drawn in the investigation of medications that can improve cardiac remodeling and long-term clinical outcomes. Currently, a routine strategy of medical therapy after the TAVI procedure is not recommended due to insufficient evidence supporting the benefits. Previous studies found that the use of RASi may have the potential to prevent or reverse cardiac remodeling and improve prognosis with less inconsistent results [8–10, 13]. Further evidence from randomized control trials is warranted to clarify the effect of RASi in cardiac remodeling and long-term prognosis. Regarding this concern, we designed this randomized control trial to investigate the effect of the prescription of RASi, specifically fosinopril, after TAVI on cardiac remodeling, clinical outcomes, and quality of life. We also summarized other randomized trials regarding this topic (Table 2). Similarly, another study in Europe, the RAS blockade after TAVI (RASTAVI) study (http://www.ClinicalTrials.gov NCT03201185), which was designed to address whether the prescription of ramipril in postoperative AS patients can be beneficial is still ongoing [17]. Together, these studies will provide further evidence regarding medical therapy for AS patients who underwent TAVI, and our study will specifically fill the gap in the Chinese population.

**Table 2:** Summary of randomized trials that investigated the effect of renin-angiotensin system blockade therapy after aortic valve replacement for severe aortic stenosis patients

Author, year	Region	Study population	Study design	Registration	Sample size^a^	Interventions	Comparison	Primary outcomes	Status
Dahl et al., 2010 [8]	Denmark, single center	Post SAVR	Randomized, open-label, blinded endpoint	NCT00294775	61:64	Candesartan treatment	Conventional treatment	Change in LV mass index	Completed
Amat-Santos et al., 2018 [17]	Spain, 8 centers	Post TAVI	Randomized, open-label, blinded endpoint	NCT03201185	168:168	Ramipril plus standard care	Standard care	Cardiac death or heart failure hospitalization or stroke	Recruiting
Aboyans et al. [18]	France, multicenter	Post SAVR or TAVI	Randomized, quadruple blinded	NCT03315832	55:55	Valsartan	Placebo	Changes in LV mass index	Not yet recruiting
Liao et al.	China, 7 centers	Post TAVI	Randomized, open-label, blinded endpoint	ChiCTR2100042266	100:100	Fosinopril plus standard care	Standard care	Change in LV mass index	Active, not yet recruiting

*SAVR* surgical aortic valve replacement, *LV* left ventricle, *TAVI* transcatheter aortic valve intervention

^a^Number of patients in the intervention group versus the control group

## Trial status

The recruitment of this study will be started in September 2021, and the last participant will be included in September 2023 anticipatedly.

## Supplementary Information

**Additional file 1: Table 1.** Study expected enrollment from seven medical centers in China.

## Abbreviations

6MWT: Six-minute walk test
AS: Aortic stenosis
CMR: Cardiac magnetic resonance
DSMC: Data and safety monitoring committee
KCCQ: Kansas City Cardiomyopathy Questionnaire
LV: Left ventricular
LVEF: Left ventricular ejection fraction
NYHA: New York Heart Association
RASi: Renin-angiotensin system inhibitors
SAVR: Surgical aortic valve replacement
TAVI: Transcatheter aortic valve implantation
