# Supplementary material for: Angiotensin-converting enzyme inhibitor for post-transcatheter aortic valve implantation patients: study protocol for a multicenter randomized, open-label blinded endpoint control trial
Source: Trials. 2021 Jul 18;22:462. doi: 10.1186/s13063-021-05411-5 (PMC8286566; doi:10.1186/s13063-021-05411-5)
Supplement: Supplementary file 1 — Additional file 1: Table 1. Study expected enrollment from seven medical centers in China. [file 13063_2021_5411_MOESM1_ESM.docx]

**Supplements**

**Table 1 Study expected enrollment from seven medical centers in China**

| **Medical Center** | **City** | **Expected number of patients enrolled** |
| --- | --- | --- |
| West China hospital | Chengdu | 60 |
| General hospital of Shenyang military region | Shenyang | 30 |
| Second Xiangya hospital of central south university | Changsha | 30 |
| Second affiliated hospital of the army medical university-Xinqiao hospital | Chongqing | 20 |
| First affiliated hospital of Xinjiang medical university | Ürümq | 20 |
| Qingdao university affiliated hospital | Qingdao | 20 |
| Fujian provincial hospital | Fuzhou | 20 |
